# Anticoccidial and Antioxidant Activities of an Ethanolic Extract of *Teucrium polium* Leaves on *Eimeria papillate*-Infected Mice

**DOI:** 10.3390/vetsci11070314

**Published:** 2024-07-14

**Authors:** Saleh Maodaa, Esam M. Al-Shaebi, Rewaida Abdel-Gaber, Afaf Alatawi, Sarah Alawwad, Dalal Alhomoud, Saleh Al-Quraishy

**Affiliations:** 1 Department of Zoology, College of Science, King Saud University, P.O. Box 2455, Riyadh 11451, Saudi Arabia; 2 Department of Food Science and Nutrition, College of Food and Agricultural Science, King Saud University, P.O. Box 2460, Riyadh 11451, Saudi Arabia

**Keywords:** coccidiosis, antioxidant, *Eimeria papillata*, *Teucrium polium*

## Abstract

**Simple Summary:**

Coccidiosis is an infectious disease that mainly affects cattle, chickens, goats, and sheep. Its source is the genus Eimeria, which produces coccidian protozoans that cause intestinal damage to their hosts. The oral intake of eimerian oocysts, which release infectious sporozoites within the colon, is the first step in the infection process. Eventually, oocysts are released with feces after they successively enter intestinal epithelial cells, where asexual multiplication occurs. Due to the widespread drug resistance brought on by the widespread use of currently available anticoccidial medicines, research into novel therapeutic approaches is being conducted. Various substances obtained from plants have shown promising and optimal anticoccidial as well as extra-therapeutic effects, in addition to other options. In Saudi Arabia, natural sources have been examined as potential controls for murine coccidiosis. Among these sources is *Teucrium polium*. Due to the aforementioned characteristics, the objective of this study was to examine *T. polium* leaf extract’s anticoccidial and antioxidant effects in *Eimeria papillata*-infected mice.

**Abstract:**

*Eimeria* spp. are responsible for the economic loss of both domestic and wild animals due to coccidiosis, the most common parasitic disease. The resistance to currently available drugs used to treat coccidiosis has been proven. Medicinal plants that contain physiologically active phytochemicals have been widely used in traditional medicine. *Teucrium polium* leaf extract (TPLE) has been shown to exhibit pharmacological, antioxidant, and anticoccidial properties in different experiments. Here, our investigation focused on how *T. polium* leaf extract affected the way that *Eimeria papillate* caused intestinal injury in mice. Thirty-five male Swiss albino mice were divided into seven groups, as follows: group I: untreated and uninfected (negative control); group II: uninfected, treated group with TPLE (150 mg/kg b.w); and group III: infected untreated (positive control). Groups III–VII were orally administered 10^3^ sporulated *E. papillata* oocysts. A total of 60 min after infection, groups IV–VI were treated for five successive days with 50, 150, and 250 mg/kg b.w TPLE, respectively, while group VII was treated with amprolium (120 mg/kg b.w.). The mice had been euthanized on the fifth day post-infection, and the jejunum tissues were prepared for histology and oxidative stress studies. A total of 150 mg/kg of TPLE was the most effective dosage, significantly decreasing oocyst output by about 80.5%, accompanied by a significant reduction in the number of developmental parasitic phases in jejunal sections. In addition, the decrease in the number of goblet cells in the jejuna of mice raised after treatment. Also, TPLE greatly diminished the body weight loss of infected mice. Moreover, our research proved that TPLE reduced oxidative damage due to *E. papillata* infection via decreasing intestinal malondialdehyde (MDA) and nitric oxide (NO) levels and increasing reduced superoxide dismutase (SOD) and glutathione (GSH) levels. These results demonstrated that TPLE had potent anticoccidial properties. TPE’s efficacy as a natural antioxidant has also been demonstrated in reducing oxidative stress and enhancing antioxidant systems to mitigate biochemical and histological changes in the jejunum caused by *E. papillata*.

## 1. Introduction

Coccidiosis, an infectious disease particularly affecting cattle, sheep, rabbits, goats and poultry, is caused by coccidian protozoans from the *Eimeria* genus that target the gastrointestinal tract of their hosts [1]. Infection initiates with the oral uptake of eimerian oocysts, which release infectious sporozoites inside the intestine. These sequentially invade primarily intestinal epithelial cells, where asexual multiplication takes place before oocysts are finally expelled with feces [2].

Owing to the quick rate of reproduction of these parasites in the digestive system, the body may encounter localized or systemic effects such as tissue damage, oxidative stress, and a significant inflammatory response. Dehydration, bleeding, reduced weight gain, decreased feed intake and nutritional absorption, and an elevated susceptibility to other infections could result from coccidiosis [3].

Al-Quraishy et al. [4] said that mice infected with *E. papillata* provide a suitable model for studying animal coccidiosis as it develops inside the cells of the small intestine in mice. While there are several anticoccidial therapies available to treat coccidiosis (such as decoquinate, toltrazuril, amprolium, diclazuril, and sulfonamide), frequent use of these medications has caused toxicity and resistance to develop. Drug resistance is a problem that has motivated many researchers to seek out alternate methods for treating coccidiosis [5]. Recent research, employing natural plant sources, offers a side-effect-free therapeutic approach and may be advantageous in treating a variety of parasitic diseases [6].

Various compounds derived from plants have demonstrated positive and ideal anticoccidial and extra-therapeutic effects, among other available alternatives. Natural sources have been tested as substitute controls for murine coccidiosis in Saudi Arabia. These sources include *Allium sativum*, *Punica granatum*, *Phoenix dactylifera*, *Ziziphus spina-christi*, *Morus nigra*, *Salvadora persica*, *Zingiber ofcinale*, and *Azadirachta indica* [7].

*Teucrium polium* is a wild-growing blooming plant found throughout Europe and southwestern Asia, as well as in the Mediterranean region [8]. This medicinal plant belongs to the Lamiaceae family, which includes species with exploitable antioxidant potential [9].

Numerous studies have documented the biological activities of *T. polium*, including its anti-inflammatory, anti-bacterial, antinociceptive, anti-hypertensive, anti-rheumatoid, hypolipidemic, hypoglycemic [10], antioxidant, anticancer [11], antibacterial [12], antiviral [13], and antiparasitic effects against *Acanthamoeba castellanii* [14]. The total flavonoids, phenolic contents, Fourier-transform infrared spectroscopy (FT-IR) results, and DPPH free radical scavenging assay results were analyzed in *T. polium* leaf extract used in this experiment [9]. Additionally, the extract was thoroughly examined in this work using HPLC–UV detection with the aim of identifying certain phenolic chemicals included in the extracts. Eleven bioactive components (myricetin, quercetin, apigenin, naringenin, luteolin 7-o-glucoside, caffeic acid, ellagic acid, rutin, and chlorogenic acid) have been identified qualitatively in the ethanolic extract.

Owing to the formerly mentioned properties, the goal of this research was to investigate the anticoccidial and antioxidant effects of *T. polium* leaf extract in infected mice with *E. papillata*.

## 2. Material and Methods

### 2.1. Experimental Animals

In the current study, thirty-five adult male Swiss albino mice of 8–10 weeks of age and 30–35 g in weight were obtained from the Department of Animal Production, College of Science, King Saud University. Mice were maintained in well-aerated cages under specific pathogen-free conditions under 12 h light/12 h dark cycles and controlled temperature (23 ± 5 °C). Animals had access to a standard pellet diet and tap water ad libitum and were acclimated over seven days prior to the start of the experiment. 

#### Ethical Approval

The study was conducted in compliance with the Kingdom of Saudi Arabia guidelines for the use of animals (King Saud University’s Ethics Committee, KSU-SE-23-56).

### 2.2. Extract Preparation and Plant Collection

*T. polium* leaves were obtained from Al-Badyah Tabuk, Saudi Arabia, in May 2022, with a position of 27°45′59.5” N, 36°31′48.8” E, 80 km south of Tabuk. The plant was identified by a specialist at the herbarium (Botany Department, Science College, King Saud University, Riyadh, Saudi Arabia). The leaf extract was prepared following the method of Qabaha et al. [15] with some adjustments. After being allowed to air dry, the leaves were powdered. The obtained powder was subjected to a cold maceration extraction technique using an ethanol (50%) solvent system for 24 h. The ethanolic extract was filtered and concentrated by a rotary evaporator under pressure and at a temperature of 50 °C, then collected and kept in sealed bottles at −20 °C.

### 2.3. Infection and Treatment

The study utilized Mus musculus laboratory mice to investigate the passage of *E. papillata* oocysts. Unsporulated oocysts were isolated from feces and allowed to sporulate in a 2.5% (*w*/*v*) potassium dichromate solution. Following sporulation, the oocysts were rinsed with phosphate buffer solution for further use in the experiment.

Seven groups of mice, each comprising five individuals, were established for the study:

Group I: Untreated and uninfected mice (negative control).

Group II: Uninfected mice receiving 150 mg/kg of TPLE orally for five consecutive days.

Group III: Infected but untreated mice (positive control).

Groups IV, V, and VI: Infected mice treated with TPLE at doses of 50, 150, and 250 mg/kg, respectively.

Group VII: Infected mice treated with 120 mg/kg of amprolium.

Groups III to VII were orally inoculated with approximately 10^3^ sporulated *E. papillata* oocysts in 100 µL of saline. Sixty minutes post-infection, groups IV, V, and VI were treated with the respective doses of TPLE, and group VII with amprolium, for five consecutive days.

Weight changes in the mice were recorded on days 0 and 5, following the methodology of Al-Quraishy et al. [16]. Fecal pellets from groups III to VII were gathered on the fifth day post-infection to quantify the total number of shed oocysts, using the method described by Schito et al. [17]. Additionally, the following formula was used to determine the suppression (%) of oocyst shedding: 100— (oocyst output in the treated group/oocyst output in the infected group) × 100. All mice were euthanized, and their jejuna were harvested and stored at −80 °C for subsequent experimental analysis.

### 2.4. Histopathological and Histochemical Investigations

Immediately after extraction, small jejuna fragments were paraffin fixed and stored in 10% neutral buffered formalin. To detect parasite stages, tissue sections (5 µm thick) were stained with hematoxylin and eosin. Alcian-blue-stained sections were then utilized to establish goblet cells. The number of *Eimeria* parasitic stages and goblet cells were counted in ten well-oriented villus–crypt units (VCUs).

### 2.5. Biochemical Analysis

In an ice-cold PBS solution, jejunum pieces were weighed and homogenized; 5000× *g* centrifugation was performed on the mixture for 15 min at 4 °C.

The intestinal homogenate’s NO assay was carried out corresponding to the method described by Berkels et al. [18]. The nitrous acid that was produced in an acidic medium with nitrite oxidized the sulfanilamide, which was subsequently combined with N-(1-naphthyl) ethylenediamine; because of this, an azo dye of an intense reddish purple color appeared. At 540 nm, the resulting color was measured. By heating the jejunum homogenate in a boiling water bath for 30 min and adding 1 milliliter each of trichloroacetic acid (10%) and thiobarbituric acid (TBA) (0.67%), the amount of lipid peroxidation in the jejunum homogenate was determined. Malondialdehyde (MDA) equivalents were used as a measure of the thiobarbituric-acid-reactive molecules that were detected by measuring the absorbance at 535 nm [19].

Using Ellman’s reagent (5,5′ dithiobis (2-nitrobenzoic acid), GSH is lowered in this procedure to get a yellow compound. GSH concentration and chromogen absorbance, which were determined at 405 nm, are directly related [20]. The method of Nishikimi et al. [21] was used to measure the homogenate’s superoxide dismutase (SOD) activity. The experiment relies on the enzyme’s ability to stop phenazine methosulphate, which is visible at 560 nm, from reducing the nitroblue tetrazolium dye.

### 2.6. Statistical Analysis

One-way analysis of variance (ANOVA) was used to analyze our data and run statistical comparisons between the groups using the SPSS (version 20) statistical programme (SPSS Inc., Chicago, IL, USA). Results were presented as the mean ± standard error of the mean, with values of *p* > 0.05 considered statistically insignificant, while those of *p* < 0.05 were considered statistically significant.

## 3. Results

### 3.1. Effect of TPLE Treatment on Fecal Oocyst Output and Body Weight

**Figure 1:**
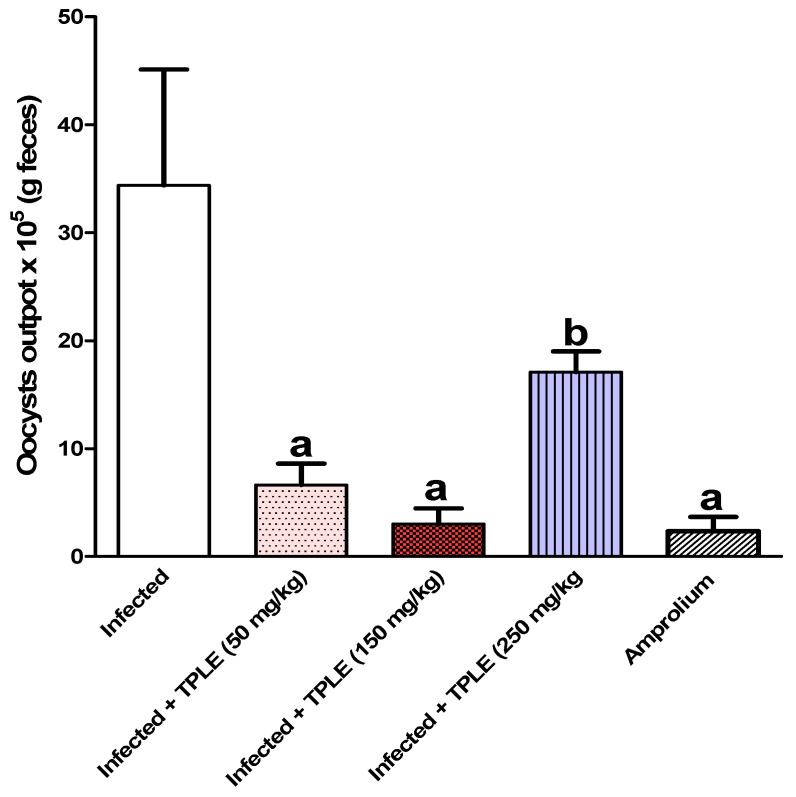
The oocyst output of mice on day 5 post-infection (p.i.) with 10^3^ sporulated *E. papillata* oocysts is presented as mean ± SEM (n = 5). ^a^ *p* ≤ 0.001 versus the infected untreated group, and ^b^ *p* ≤ 0.05 versus the infected untreated group.

**Figure 2:**
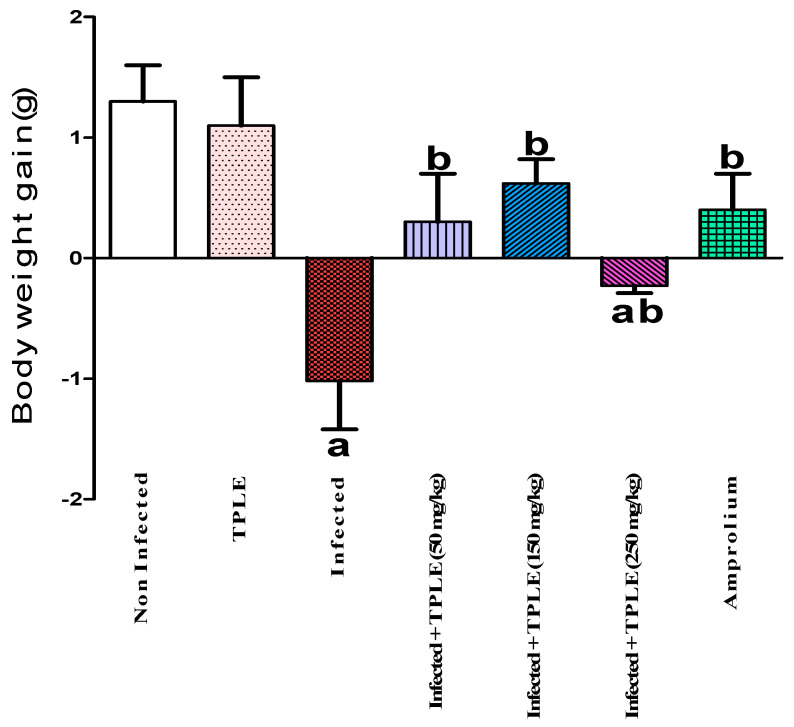
*T. polium* leaf extract improved weight loss because of infection with *E. papillata*. Values are mean ± SEM. ^a^ *p* ≤ 0.001 as compared with uninfected control, and ^b^ *p* ≤ 0.01 versus the infected untreated group (n = 5).

**Figure 3:**
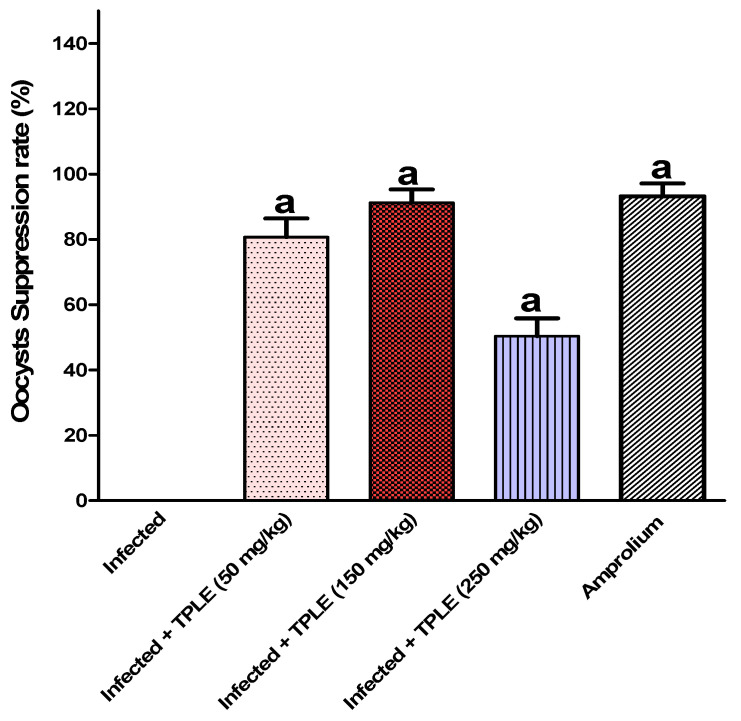
Rate of suppression of *E. papillata* oocysts in the infected treated mice and those infected with three different doses of TPLE, as well as infected amprolium-treated mice on day 5 p.i. A significant difference was noted as compared with infected untreated mice (*p* ≤ 0.001). All values are expressed as mean ± SEM (n = 5).

The study results demonstrated the release of oocysts in fecal pellets, with the highest level observed in the infected group 5 days post-infection at approximately 34.394 × 10^5^ ± 10.72 × 10^5^ oocysts per gram feces. Injection of infected mice with various doses of TPLE produced a significant decrease in the total number of ejected oocysts, especially after oral dosing with 150 mg/kg TPLE (3.027 × 10^5^ ± 1.4231 × 10^5^ oocysts/g feces), as compared with the group treated with the reference drug amprolium (2.3352 × 10^5^ ± 1.3356 × 10^5^ oocysts/g feces). Though the other two doses exhibit a considerable decrease, 50 mg/kg of extract (6.6308 × 10^5^ ± 1.9803 × 10^5^ oocysts/g feces) 250 mg/kg of extract (17.087 × 10^5^ ± 1.9102 × 10^5^ oocysts/g feces) also showed a reduction (Figure 1). At the same time, the average weight of mice was significantly reduced (−1.02 ± 0.4 g) in the infected untreated group compared with the uninfected control group, while the latter mice who fed normally without infection showed an average rise in body weight (1.3 ± 0.3 g). Interestingly, administering different doses of leaf extract treatment to *Eimeria-infected* mice changed their body weight gain/gm, especially with 150 mg/kg TPLE (0.62 ± 0.2 g); however, other remaining groups induced changes with 0.3 ± 0.4 g, 0.4 ± 0.3 g, and 1.1 ± 0.4 g for the infected + 50 mg/kg of extract group, amprolium-treated group, and uninfected TPLE-treated group, respectively, compared to the uninfected untreated group and contrary to the infected + 250 mg/kg TPLE-treated group (−0.23 ± 0.06 g) (Figure 2). As a result, one dose was chosen in the subsequent investigations, which was 150 mg/kg, as this dose was the most effective in lowering the fecal oocyst output, oocyst suppression, and enhanced body weight gain. Similarly, all different doses of *Teucrium polium* leaf extract treatment as well as amprolium were significantly (*p* < 0.001) able to suppress the oocyst output compared with the infected group (Figure 3).

### 3.2. Histological Observation

**Figure 4:**
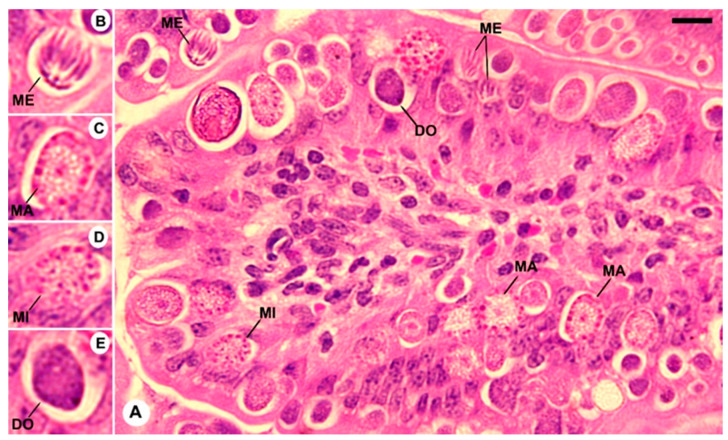
The evolutionary stages of *E. papillata* in the jejunum of infected mice on day 5 post-infection (p.i.) are illustrated. (**A**) Various developmental stages are observed, including (**B**) meronts (ME), (**C**) macrogamonts (MA), (**D**) microgamonts (MI), and (**E**) developing oocysts (DO). Tissue sections were stained with hematoxylin and eosin (H&E). The scale bar represents 12.5 μm.

**Figure 5:**
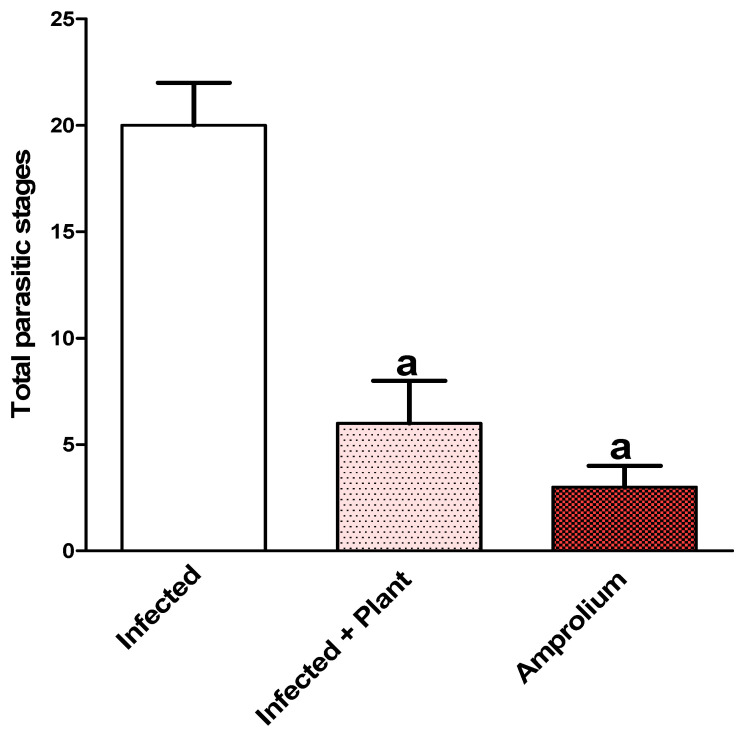
Histograms showed that 150 mg/kg TPLE treatment induced a significant reduction in the number of *Eimeria* stages in infected mouse jejuna as well as amprolium; ^a^ *p* ≤ 0.001 as compared with infected untreated mice. All values are expressed as mean ± SEM (n = 5).

**Figure 6:**
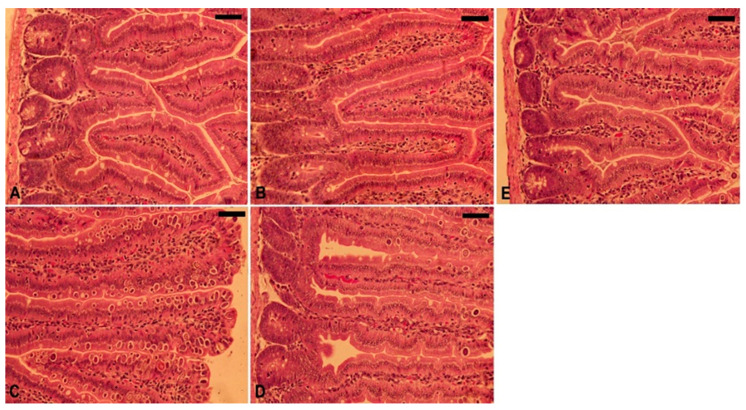
Histological alterations in jejunum tissue of mice during *E. papillata* infection and after treatment with TPLE: (**A**) uninfected group, (**B**) TPLE group, (**C**) infected group, (**D**) infected+ TPLE (150 mg/kg) group, and (**E**) amp group; scale bar = 50 μm.

Upon experimental infection of the mice with *E. papillata* oocysts, several stages of the parasite developed in the jejunal epithelial cells (Figure 4). Comparing the 150 mg/kg TPLE group to the infected group, there was a significant (*p* < 0.001) decrease in the number of parasite stages per ten villus–crypt units (Figure 5 and Figure 6). 

### 3.3. Effect of TPLE Treatment on Intestinal Goblet Cells

**Figure 7:**
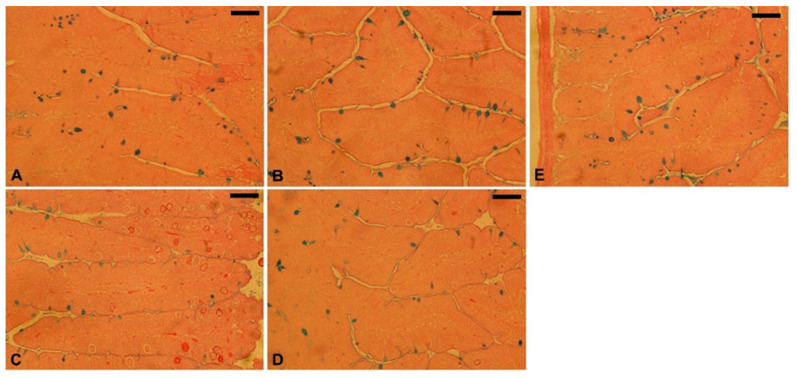
Jejunal goblet cells stained with Alcian blue: (**A**) uninfected group, (**B**) TPLE-treated group, (**C**) infected group, (**D**,**E**) infected treated groups with TPLE (150 mg/kg) and Amp, respectively; scale bar = 50 μm.

**Figure 8:**
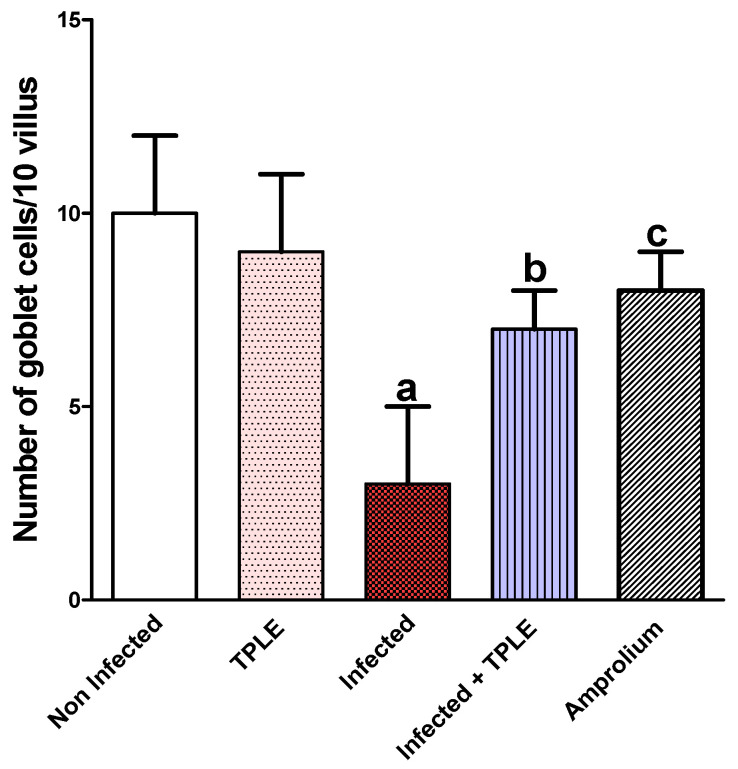
Statistical analysis illustrates significant decline in goblet cell numbers in the infected group compared to the uninfected control group. However, TPLE and amprolium treatment increased the number of goblet cells significantly. All values are expressed as mean ± SEM (n = 5); ^a^ *p* < 0.001 versus the uninfected control group, ^b^ *p* < 0.05 versus the infected group, and ^c^ *p* < 0.01 versus the infected group.

The goblet cell number was significantly reduced in the mice jejuna owing to *E. papillata* infection in comparison with the uninfected control group (10 ± 2). However, treatment with 150 mg/kg TPLE resulted in a considerable increase (7 ± 1) in the goblet cell number compared with the infected group (3 ± 2). The amprolium-treated group had an elevated number of goblet cells (8 ± 1) compared to the infected group. However, the uninfected TPLE-treated group had an enhanced goblet cell number (9 ± 2) (Figure 7 and Figure 8).

### 3.4. Effect of TPLE Treatment on Oxidative Stress in Jejunal Tissue

**Figure 9:**
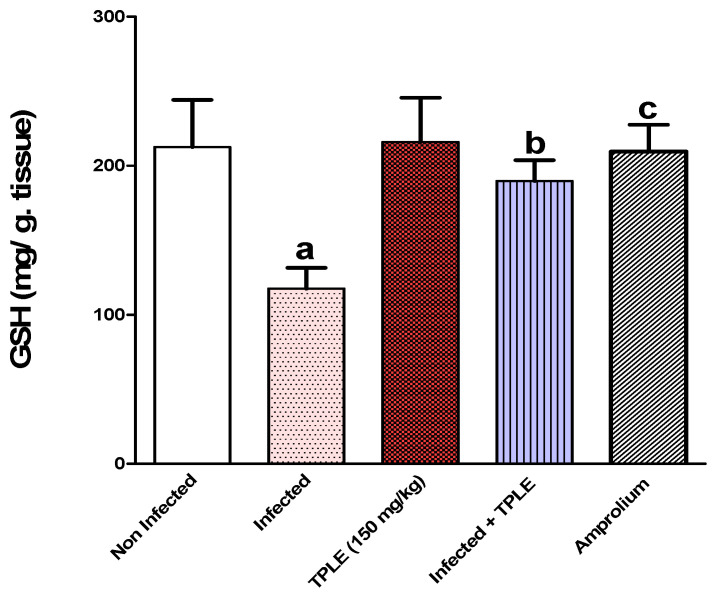
Effect of *T. polium* leaves on the value of glutathione (GSH) in mice infected with *E. papillate*; ^a^ *p* < 0.01 versus the uninfected control group, ^b^ *p* < 0.05 versus the infected group, and ^c^ *p* < 0.01 versus the infected group. All values are expressed as mean ± SEM (n = 5).

**Figure 10:**
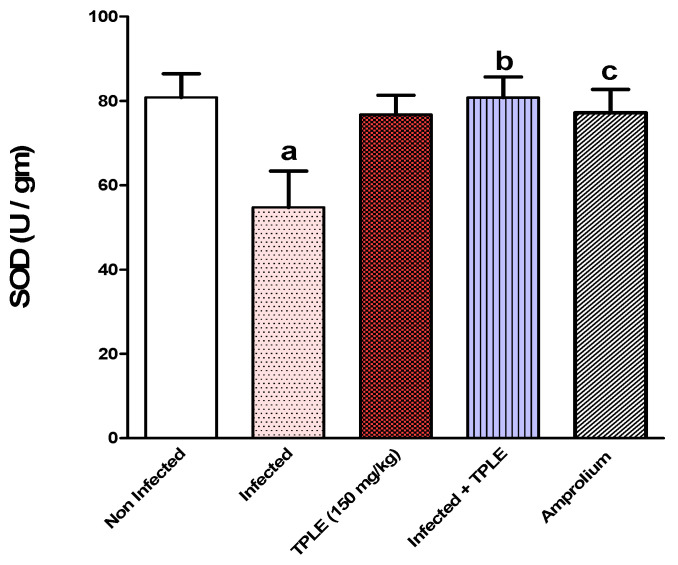
TPLE elevates jejunum super oxide dismutase (SOD) level in *E. papillate*-infected mice; ^a^ *p* < 0.01 versus the uninfected control group, ^b^ *p* < 0.01 versus the infected group, and ^c^ *p* < 0.05 versus the infected group. All values are expressed as mean ± SEM (n = 5).

In the *Eimeria*-infected group, the reduced glutathione (GSH) level was significantly lower (*p* < 0.01) than in the uninfected group. In contrast, GSH levels were elevated in mice treated with 150 mg/kg of TPLE and amprolium (*p* < 0.05 and *p* < 0.01, respectively), compared to the infected untreated group (Figure 9). Similarly, a significant reduction (*p* < 0.01) in superoxide dismutase (SOD) has been observed in the infected untreated group compared with the uninfected control group. However, there was a considerable increase in the SOD levels of both infected groups treated with 150 mg/kg of plant extract and the amprolium-treated group (*p* < 0.01 and *p* < 0.05) compared with the infected untreated group (Figure 10). 

**Figure 11:**
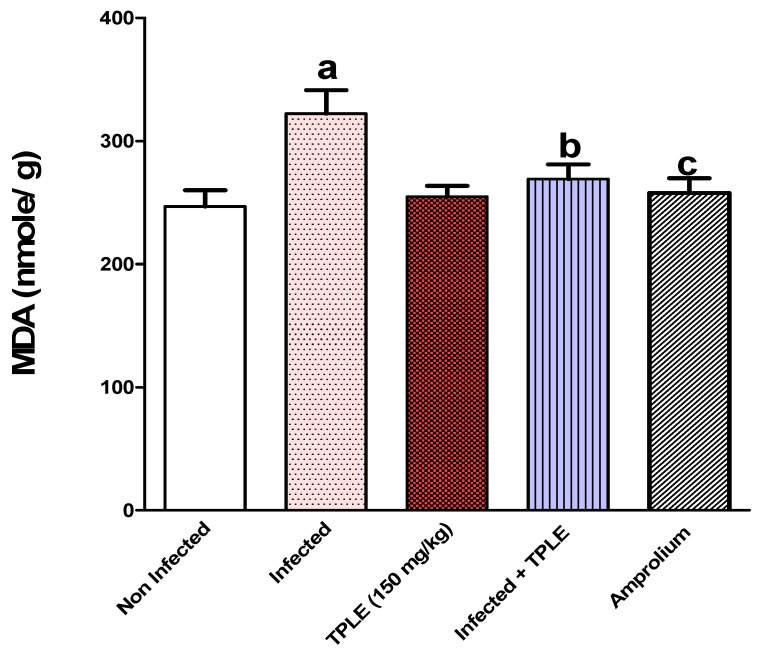
*T. polium* leaf extract improved the malondialdehyde (MDA) level of mice infected with *E. papillate*; ^a^ *p* < 0.001 versus the uninfected control group, ^b^ *p* < 0.05 versus the infected group, and ^c^ *p* < 0.01 versus the infected group. All values are expressed as mean ± SEM (n = 5).

**Figure 12:**
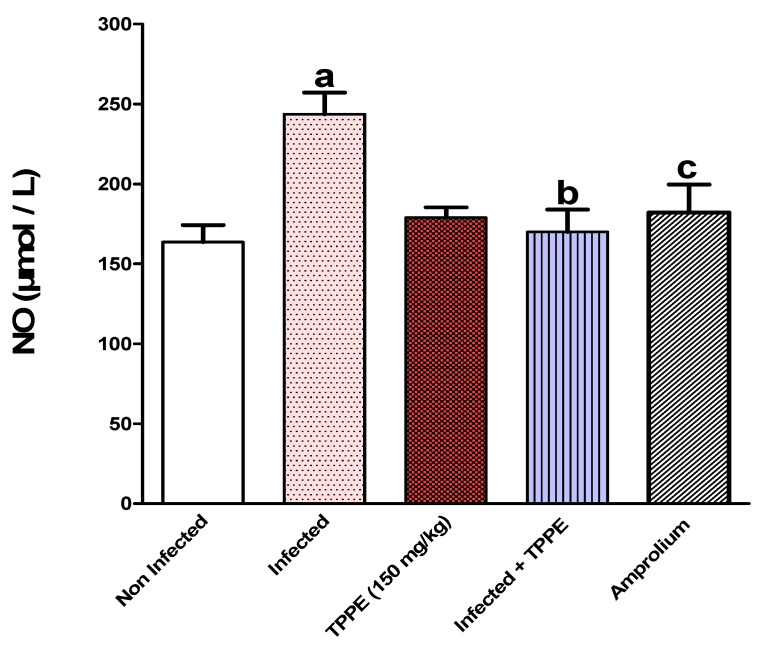
TPLE decreased jejunal nitric oxide (NO) level in *E.-papillate*-infected mice; ^a^ *p* < 0.001 versus the uninfected control group, ^b^ *p* < 0.001 versus the infected group, and ^c^ *p* < 0.01 versus the infected group. All values are expressed as mean ± SEM (n = 5).

Additionally, infection was associated with a highly significant rise (*p* < 0.001) in the levels of malondialdehyde (MDA) (Figure 11) and nitric oxide (NO) (Figure 12) compared with the uninfected control group.

Oral dosing with 150 mg/kg of TPLE and amprolium significantly reduced the levels of MDA and NO. The reductions in MDA levels were significant at *p* < 0.05 and *p* < 0.01, while the reductions in NO levels were significant at *p* < 0.001 and *p* < 0.01, compared to the *Eimeria*-infected group (Figure 11 and Figure 12).

## 4. Discussion

Coccidiosis is the most frequent infectious disease affecting animals and birds that has considerable negative financial effects through mortality, lowered weight gains, and bad feed efficiencies [22]. The recommended strategy for managing coccidiosis involves the use of accessible anticoccidial medications, However, the continued abuse of these medications has resulted in drug resistance [23]. Therefore, major efforts are currently being made to develop herbal remedies with potent anticoccidial effects as a different strategy for control of the disease [24]. The results of the current study reveal the potent anti-eimerial activity of *T. polium* extract (TPLE) against mouse intestinal *E. papillata* infection. This study demonstrated that TPLE is an effective therapy for *E. papillata* infection in mice, as evidenced by its ability to decrease oocyst output. Such a decreased output suggests that leaf extract impedes the host intestinal cells from developing intracellular *Eimeria* stages before the oocyst forms and is finally released via the feces. Al-Shaebi et al. [8] have previously reported that *T. polium* exhibits anticoccidial activities. This effect may be due to the bioactive phytochemical ingredients present in the extract, such as essential oils, tannins, flavonoids, sterols, saponins [25,26,27], diterpenoids, and iridoids. Saponins derived from plant sources are believed to have several therapeutic properties, such as antibacterial, anti-inflammatory, antidiabetic, and anticancer effects [28,29,30]. TPLE anticoccidial efficacy, demonstrated by the high suppression of the percentage oocyst rate, may stem from its saponin component, which has anticoccidial properties and inhibits protozoan growth by reacting with cholesterol on the parasite cell membrane to cause parasitic death [31]. Moreover, *Acanthamoeba castellani* trophozoites and cysts tested in vitro with *T. polium* and *Teucrium chamaedrys* methanolic extract could significantly decrease in a dose- and time-dependent manner. The extract’s amoebicidal effect could be caused by a particular interaction between the active phytochemicals and the parasite membranes, as well as by a more efficient way of entering the parasites via membrane channels [14].

Presently, intestinal tissue damage results from *Eimeria* infections in mice because of the parasites’ development stages, particularly due to merozoites bursting out of the gut cells and destroying other intestinal cells. The total count of parasite stages in the jejunum was significantly reduced because of the TPP extract’s anti-eimerial effect. *Persea americana* extract (PAE) was reported to induce the same effect [7].

Numerous external pathological alterations, including severe diarrhea, overall weakness, anorexia, and significant weight loss, were linked to infection. This significant weight reduction could be caused by many factors, including reduced daily food and water intake [32]; decreased energy production within infected tissues, which results in decreased nutrient active transport; and consequently, malabsorption, and disturbance of metabolic status.

In the present study, treatment with TPLE was able to restore the amount of goblet cells in the infected mice’s intestines, unlike in the infected untreated mice. Goblet cells are immunocompetent, significant intestinal cells that can secrete mucus, which serves as a barrier of defense, according to Linh [33]. Numerous potential stem cells can be found in the jejunal villi. A decline in goblet cells could be a sign that the population of stem cells has been damaged by the parasite [34]. The host’s capacity to manage the infection’s spread or penetration into the surrounding epithelium in an *Eimeria*-infected host may be affected by the change in goblet cells [35]. Abdel-Latif et al. [36] claim that mucus traps parasites and limits their ability to move and feed.

According to our research, oxidative jejunal damage due to *Eimeria* infection in mice is associated with a decrease in antioxidant enzymes such as GSH and SOD. These enzymes are essential for preventing free-radical damage to the animal’s body when it is infected with Eimeria, whereas their decrease causes damaged DNA and cellular membranes, elevated protein oxidation, lipid peroxidation, changed intracellular stability, and the induction of cell death. Reactive oxygen species levels have increased, causing this irreversible damage [37], together with nitric oxide (NO) and malondialdehyde (MDA), which were produced as part of the host’s cellular immunological response to the *Eimeria* infection and were intricately linked to the pathophysiology of intestinal coccidiosis. Giving TPLE to mice with *E. Papillata* infection could reduce oxidative stress in the infected jejunum, supported here by an impairment of *Eimeria*-induced increases in both NO and MDA as well as elevated SOD and GSH levels. Owing to these findings, TPLE has considerable antioxidant activity, and this is in accordance with the findings of Ljubuncic et al. [38], who claim that TPE contains free-radical flavonoid scavenging properties, providing protection against oxidative damage. In this regard, *T. polium* extract prevented the oxidation of AAPH-induced plasma oxidation, β-carotene, and Fe^2+^-induced lipid peroxidation in rat liver homogenates, besides scavenging OH• and O^−2^; This resulted in bound free iron and tended to increase intracellular GSH levels resulting in a reduce in the GSSG/GSH ratio.

## 5. Conclusions

Our findings have proved that TPLE, at a dose of (150 mg/kg body weight), can protect mice against *E. papillate* infection as it reduces oocyst output and intestinal developmental stages, as well as improves the standardization of goblet cell populations. Moreover, the effect of TPLE comes from an increase in the intestinal levels of SOD and GSH, along with a decrease in NO and MDA. TPLE, once incorporated into an animal’s regular diet, protects host tissues against impairments caused by a variety of pathogenic diseases. Further research is required to determine the extract’s anti-inflammatory activity, degree of apoptosis, and mode of action on the parasite and host.

## Data Availability

All the datasets generated or analyzed during this study are included in this published article.
